# Bis{2,6-bis­[(2-hy­droxy-5-methyl­phen­yl)imino­meth­yl]pyridine} monohydrate

**DOI:** 10.1107/S1600536811045399

**Published:** 2011-11-05

**Authors:** Muhammet Kose, Vickie McKee

**Affiliations:** a Department of Chemistry, Loughborough University, Loughborough LE11 3TU, England

## Abstract

The title compound, 2C_21_H_19_N_3_O_2_·H_2_O, was synthesized by a Schiff base condensation of 2,6-diformyl­pyridine with 2-amino-4-methyl­phenol in ethanol. In the crystal, two mol­ecules of 2,6-bis­[(2-hy­droxy-5-methyl­phen­yl)imino­meth­yl]pyridine dimer­ize *via* hydrogen bonding to a water mol­ecule, which lies on a twofold axis. There are also intra­molecular phenol–imine hydrogen bonds. The dimers are further linked *via* π–π (phen­yl–pyridine) [centroid–centroid distance = 3.707 (2) Å] and π–π edge-to-edge [3.392 (2) Å] inter­actions. The dihedral angles between the central ring and the two pendant rings are 11.46 (8) and 2.06 (8)° while the pendant rings make a dihedral angle of 10.14 (8)°.

## Related literature

For related structures, see: Gonzalez *et al.* (2008 ▶); Sun *et al.* (2006 ▶).
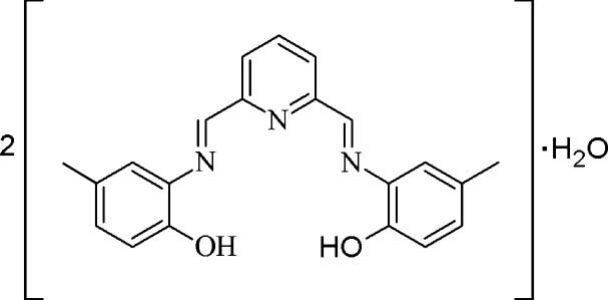


## Experimental

### 

#### Crystal data

2C_21_H_19_N_3_O_2_·H_2_O
*M* *_r_* = 708.80
Monoclinic, 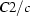

*a* = 23.8510 (13) Å
*b* = 12.9688 (7) Å
*c* = 12.3835 (7) Å
β = 114.077 (1)°
*V* = 3497.2 (3) Å^3^
*Z* = 4
Mo *K*α radiation
μ = 0.09 mm^−1^
*T* = 150 K
0.60 × 0.28 × 0.11 mm

#### Data collection

Bruker APEXII CCD diffractometer
Absorption correction: multi-scan (*SADABS*; Sheldrick, 2008 ▶) *T*_min_ = 0.947, *T*_max_ = 0.991
19364 measured reflections
5005 independent reflections
3919 reflections with *I* > 2σ(*I*)
*R*_int_ = 0.024

#### Refinement

*R*[*F* ^2^ > 2σ(*F* ^2^)] = 0.042
*wR*(*F* ^2^) = 0.124
*S* = 1.06
5005 reflections
251 parameters
H atoms treated by a mixture of independent and constrained refinement
Δρ_max_ = 0.36 e Å^−3^
Δρ_min_ = −0.23 e Å^−3^

### 

Data collection: *APEX2* (Bruker, 1998 ▶); cell refinement: *SAINT* (Bruker, 1998 ▶); data reduction: *SAINT*; program(s) used to solve structure: *SHELXS97* (Sheldrick, 2008 ▶); program(s) used to refine structure: *SHELXL97* (Sheldrick, 2008 ▶); molecular graphics: *SHELXTL* (Sheldrick, 2008 ▶); software used to prepare material for publication: *SHELXTL*.

## Supplementary Material

Crystal structure: contains datablock(s) I, global. DOI: 10.1107/S1600536811045399/aa2032sup1.cif

Structure factors: contains datablock(s) I. DOI: 10.1107/S1600536811045399/aa2032Isup2.hkl

Supplementary material file. DOI: 10.1107/S1600536811045399/aa2032Isup3.cml

Additional supplementary materials:  crystallographic information; 3D view; checkCIF report

## Figures and Tables

**Table 1:** Hydrogen-bond geometry (Å, °)

*D*—H⋯*A*	*D*—H	H⋯*A*	*D*⋯*A*	*D*—H⋯*A*
O2—H2*A*⋯O3	0.836 (17)	1.960 (17)	2.7592 (12)	159.6 (15)
O2—H2*A*⋯N3	0.836 (17)	2.347 (15)	2.7624 (12)	111.3 (12)
O1—H1*A*⋯N1	0.86 (2)	2.120 (19)	2.6722 (13)	121.7 (16)
O3—H3*D*⋯N2	0.812 (16)	2.187 (16)	2.9865 (12)	168.0 (16)
O3—H3*D*⋯N3	0.812 (16)	2.569 (16)	3.0142 (9)	115.9 (14)

**Fig. 1.**
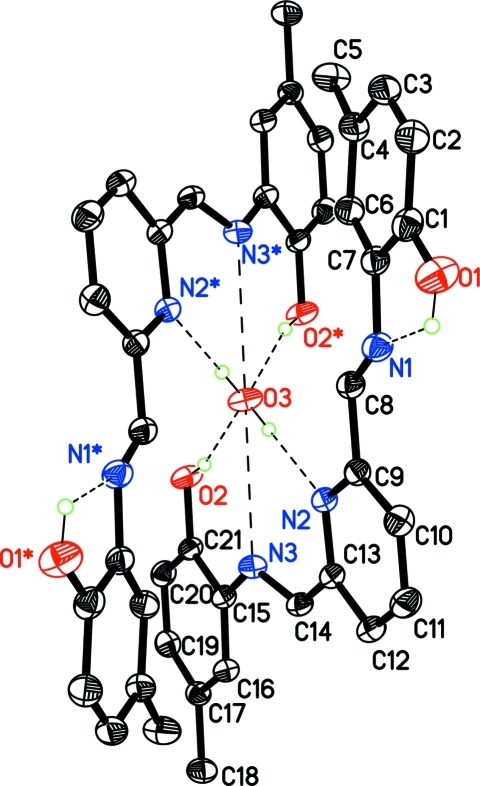
The structure of the dimeric compound, thermal ellipsoid 50% probability, hydrogen atoms bonded to carbon atoms are omitted for clarity, hydrogen bonds are shown as dash lines, symmetry operation * 1 - x, y, 1/2 - z.

**Fig. 2.**
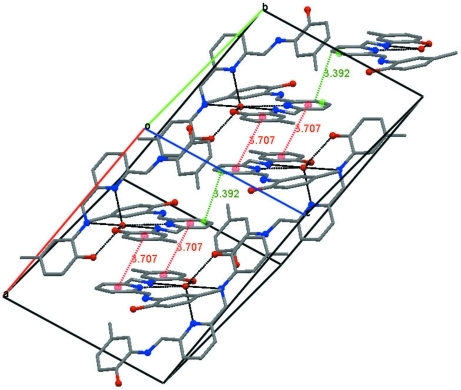
π-π (phenyl-pyridine) and π-π edge to edge interactions in the lattice.

## supplementary crystallographic information

### Comment

The molecules crystallize as dimers, assembled by hydrogen bonding around a water molecule. The water molecule sits on a twofold axis and makes four strong hydrogen bonds, as donor to the pyridine nitrogen atoms, and as acceptor from the phenol groups (Table 1). There are also weaker hydrogen bond interactions with one of the imine nitrogen atoms of each molecule. Additionally, there are phenol-imine intramolecular hydrogen bonds in each molecule. The azomethine (C8═N1 and C14═N3) linkage distances are 1.278 (14) and 1.273 (14) Å respectively and within the normal C═N values.

The geometry is *cis* at the C8═N1 imine group and *trans* at C14═N3 group. The molecule is close to planar with interplanar angles between the pyridine and phenol groups, 11.46 (8)° and 2.06 (8)° for the rings containing O1 and O2 respectively.

The hydrogen bond geometry at the water molecule is approximately tetrahedral but the angle between the two H-bonded Schiff base molecules is not close to the ideal 90°; the angle between the N2/O3/O2 plane and its equivalent under twofold rotation is 60.52 (4)°. This is possibly a consequence of the intermolecular interactions in the lattice. The Schiff base molecules show two sets of interactions with neighbouring dimeric units. On the more open face (that nearest to the H-bonded phenol of the second molecule), there is a π-π interaction involving the pyridine ring and the phenolimine group H-bonded to central water molecule. The average interplanar distance between this section and its neighbour under symmetry operation 1 - *x*, -*y*, -*z* is 3.354 (1) Å and phenol-pyridine centroid-centroid distance is 3.707 (2) Å. On the other face (partially blocked by the non-bonded phenol of the second molecule), there is a less extensive edge to edge interaction C10 to C11 of a neighbouring dimer under symmetry operation 1 - *x*, 1 - *y*, -*z* is 3.392 (2) Å. These result in columns formed by alternating π-π and edge to edge interactions extending through the lattice.

### Experimental

A solution of 2,6-diformylpyridine (0.405 g, 3 mmol) in ethanol (15 ml) was added dropwise to an ethanolic solution (30 ml) of 2-amino-4-methylphenol (0.740 g, 6 mmol). A yellow colour appeared and a precipitate was formed in a few minutes. The mixture was stirred for two hours, and then the yellow product was collected by filtration, washed with ethanol-diethylether mixture (1:1) and dried in air. Yield: 0.852 g, 80%, m.p 173–177 C°. Analysis Calc. for C_21_H_19_N_3_O_2_^.^0.5(H_2_O): C, 71.17; H, 5.69; N, 11.86%. Found: C, 71.03; H, 5.65; N, 11.70%.

### Refinement

H atoms bonded to C atoms were inserted at calculated positions with C—H distances of 0.95 and 0.99 Å for non-saturated and saturated C atoms, respectively. They were refined using a riding model with *U*_iso_(H) = 1.2*U*_eq_(C). The H-atoms bonded to O1, O2 and O3 are taken directly from the difference Fourier and were refined with temperature factors riding on the carrier atom.

### Crystal data

**Table:**

2C_21_H_19_N_3_O_2_·H_2_O	*F*(000) = 1496
*M**_r_* = 708.80	*D*_x_ = 1.346 Mg m^−^^3^
Monoclinic, *C*2/*c*	Mo *K*α radiation, λ = 0.71073 Å
Hall symbol: -C 2yc	Cell parameters from 5957 reflections
*a* = 23.8510 (13) Å	θ = 2.3–29.8°
*b* = 12.9688 (7) Å	µ = 0.09 mm^−^^1^
*c* = 12.3835 (7) Å	*T* = 150 K
β = 114.077 (1)°	Block, yellow
*V* = 3497.2 (3) Å^3^	0.60 × 0.28 × 0.11 mm
*Z* = 4	

### Data collection

**Table:**

Bruker APEXII CCD diffractometer	5005 independent reflections
Radiation source: fine-focus sealed tube	3919 reflections with *I* > 2σ(*I*)
graphite	*R*_int_ = 0.024
φ and ω scans	θ_max_ = 29.8°, θ_min_ = 1.8°
Absorption correction: multi-scan (*SADABS*; Sheldrick, 2008)	*h* = −33→33
*T*_min_ = 0.947, *T*_max_ = 0.991	*k* = −17→18
19364 measured reflections	*l* = −17→17

### Refinement

**Table:**

Refinement on *F*^2^	Primary atom site location: structure-invariant direct methods
Least-squares matrix: full	Secondary atom site location: difference Fourier map
*R*[*F*^2^ > 2σ(*F*^2^)] = 0.042	Hydrogen site location: inferred from neighbouring sites
*wR*(*F*^2^) = 0.124	H atoms treated by a mixture of independent and constrained refinement
*S* = 1.06	*w* = 1/[σ^2^(*F*_o_^2^) + (0.0642*P*)^2^ + 1.5272*P*] where *P* = (*F*_o_^2^ + 2*F*_c_^2^)/3
5005 reflections	(Δ/σ)_max_ = 0.001
251 parameters	Δρ_max_ = 0.36 e Å^−^^3^
0 restraints	Δρ_min_ = −0.23 e Å^−^^3^

### Special details

**Table:**

Geometry. All s.u.'s (except the s.u. in the dihedral angle between two l.s. planes) are estimated using the full covariance matrix. The cell s.u.'s are taken into account individually in the estimation of s.u.'s in distances, angles and torsion angles; correlations between s.u.'s in cell parameters are only used when they are defined by crystal symmetry. An approximate (isotropic) treatment of cell s.u.'s is used for estimating s.u.'s involving l.s. planes.
Refinement. Refinement of *F*^2^ against ALL reflections. The weighted *R*-factor *wR* and goodness of fit *S* are based on *F*^2^, conventional *R*-factors *R* are based on *F*, with *F* set to zero for negative *F*^2^. The threshold expression of *F*^2^ > σ(*F*^2^) is used only for calculating *R*-factors(gt) *etc*. and is not relevant to the choice of reflections for refinement. *R*-factors based on *F*^2^ are statistically about twice as large as those based on *F*, and *R*- factors based on ALL data will be even larger.

### Fractional atomic coordinates and isotropic or equivalent isotropic displacement parameters (Å^2^)

**Table:**

	*x*	*y*	*z*	*U*_iso_*/*U*_eq_	
O1	0.33231 (5)	0.57957 (7)	0.14787 (9)	0.0363 (2)	
H1A	0.3535 (9)	0.5552 (15)	0.1120 (17)	0.054*	
C1	0.32840 (6)	0.50007 (9)	0.21682 (10)	0.0263 (2)	
C2	0.29313 (6)	0.51152 (9)	0.28174 (11)	0.0295 (3)	
H2	0.2724	0.5747	0.2792	0.035*	
C3	0.28826 (5)	0.43063 (9)	0.35006 (11)	0.0285 (2)	
H3	0.2641	0.4391	0.3944	0.034*	
C4	0.31806 (5)	0.33642 (9)	0.35549 (10)	0.0259 (2)	
C5	0.30934 (6)	0.24734 (10)	0.42542 (12)	0.0344 (3)	
H5A	0.3220	0.1832	0.4000	0.052*	
H5B	0.2660	0.2426	0.4119	0.052*	
H5C	0.3344	0.2584	0.5098	0.052*	
C6	0.35360 (5)	0.32612 (9)	0.29095 (10)	0.0243 (2)	
H6	0.3744	0.2630	0.2942	0.029*	
C7	0.35935 (5)	0.40707 (9)	0.22099 (9)	0.0232 (2)	
N1	0.39157 (5)	0.40486 (7)	0.14721 (8)	0.0250 (2)	
C8	0.42258 (5)	0.32597 (9)	0.14243 (9)	0.0231 (2)	
H8	0.4261	0.2691	0.1931	0.028*	
C9	0.45300 (5)	0.32110 (8)	0.06030 (9)	0.0209 (2)	
C10	0.44553 (5)	0.39831 (9)	−0.02331 (10)	0.0250 (2)	
H10	0.4218	0.4580	−0.0268	0.030*	
C11	0.47345 (6)	0.38588 (9)	−0.10081 (10)	0.0274 (2)	
H11	0.4695	0.4372	−0.1582	0.033*	
C12	0.50726 (5)	0.29722 (9)	−0.09334 (10)	0.0251 (2)	
H12	0.5265	0.2866	−0.1461	0.030*	
C13	0.51268 (5)	0.22380 (8)	−0.00723 (9)	0.0205 (2)	
N2	0.48638 (4)	0.23536 (7)	0.06979 (8)	0.02020 (19)	
C14	0.54842 (5)	0.12966 (8)	−0.00076 (9)	0.0219 (2)	
H14	0.5582	0.1125	−0.0657	0.026*	
N3	0.56667 (4)	0.07051 (7)	0.08909 (8)	0.02191 (19)	
C15	0.60324 (5)	−0.01660 (8)	0.09325 (9)	0.0199 (2)	
C16	0.63466 (5)	−0.03133 (8)	0.02040 (9)	0.0210 (2)	
H16	0.6319	0.0200	−0.0362	0.025*	
C17	0.66968 (5)	−0.11873 (8)	0.02855 (9)	0.0218 (2)	
C18	0.70282 (5)	−0.13137 (9)	−0.05144 (10)	0.0276 (2)	
H18A	0.6833	−0.0877	−0.1215	0.041*	
H18B	0.7008	−0.2036	−0.0758	0.041*	
H18C	0.7459	−0.1109	−0.0090	0.041*	
C19	0.67339 (5)	−0.19289 (8)	0.11334 (10)	0.0242 (2)	
H19	0.6969	−0.2536	0.1200	0.029*	
C20	0.64359 (5)	−0.17978 (9)	0.18780 (10)	0.0240 (2)	
H20	0.6471	−0.2309	0.2452	0.029*	
C21	0.60848 (5)	−0.09168 (8)	0.17862 (9)	0.0212 (2)	
O2	0.57884 (4)	−0.08313 (7)	0.25167 (7)	0.02766 (19)	
H2A	0.5591 (7)	−0.0281 (13)	0.2415 (13)	0.033*	
O3	0.5000	0.07316 (9)	0.2500	0.0278 (3)	
H3D	0.4959 (8)	0.1100 (13)	0.1942 (14)	0.042*	

### Atomic displacement parameters (Å^2^)

**Table:**

	*U*^11^	*U*^22^	*U*^33^	*U*^12^	*U*^13^	*U*^23^
O1	0.0520 (6)	0.0268 (4)	0.0359 (5)	0.0110 (4)	0.0239 (4)	0.0038 (4)
C1	0.0301 (6)	0.0243 (5)	0.0224 (5)	0.0038 (4)	0.0088 (4)	−0.0028 (4)
C2	0.0304 (6)	0.0275 (6)	0.0300 (6)	0.0074 (5)	0.0119 (5)	−0.0056 (5)
C3	0.0263 (5)	0.0334 (6)	0.0272 (6)	0.0027 (5)	0.0122 (5)	−0.0071 (5)
C4	0.0242 (5)	0.0290 (6)	0.0251 (5)	0.0010 (4)	0.0106 (4)	−0.0033 (4)
C5	0.0364 (7)	0.0364 (7)	0.0386 (7)	0.0026 (5)	0.0236 (6)	0.0021 (5)
C6	0.0242 (5)	0.0247 (5)	0.0241 (5)	0.0043 (4)	0.0099 (4)	−0.0023 (4)
C7	0.0237 (5)	0.0249 (5)	0.0205 (5)	0.0026 (4)	0.0084 (4)	−0.0036 (4)
N1	0.0278 (5)	0.0258 (5)	0.0226 (5)	0.0032 (4)	0.0115 (4)	−0.0022 (4)
C8	0.0235 (5)	0.0252 (5)	0.0210 (5)	0.0021 (4)	0.0097 (4)	−0.0007 (4)
C9	0.0203 (5)	0.0229 (5)	0.0193 (5)	0.0002 (4)	0.0077 (4)	−0.0021 (4)
C10	0.0268 (5)	0.0223 (5)	0.0249 (5)	0.0021 (4)	0.0098 (4)	0.0009 (4)
C11	0.0315 (6)	0.0270 (5)	0.0241 (5)	0.0007 (4)	0.0117 (5)	0.0056 (4)
C12	0.0270 (5)	0.0302 (6)	0.0199 (5)	0.0012 (4)	0.0115 (4)	0.0021 (4)
C13	0.0207 (5)	0.0242 (5)	0.0168 (4)	0.0003 (4)	0.0079 (4)	−0.0007 (4)
N2	0.0205 (4)	0.0226 (4)	0.0182 (4)	0.0003 (3)	0.0086 (3)	−0.0012 (3)
C14	0.0232 (5)	0.0267 (5)	0.0185 (5)	0.0021 (4)	0.0110 (4)	−0.0009 (4)
N3	0.0248 (4)	0.0221 (4)	0.0224 (4)	0.0006 (3)	0.0133 (4)	−0.0004 (3)
C15	0.0224 (5)	0.0201 (5)	0.0182 (5)	0.0001 (4)	0.0093 (4)	−0.0011 (4)
C16	0.0231 (5)	0.0215 (5)	0.0203 (5)	0.0003 (4)	0.0109 (4)	0.0008 (4)
C17	0.0217 (5)	0.0225 (5)	0.0223 (5)	−0.0011 (4)	0.0102 (4)	−0.0031 (4)
C18	0.0295 (6)	0.0278 (6)	0.0307 (6)	0.0032 (5)	0.0176 (5)	−0.0011 (4)
C19	0.0235 (5)	0.0218 (5)	0.0268 (5)	0.0022 (4)	0.0097 (4)	0.0000 (4)
C20	0.0263 (5)	0.0232 (5)	0.0215 (5)	−0.0003 (4)	0.0086 (4)	0.0034 (4)
C21	0.0230 (5)	0.0246 (5)	0.0171 (5)	−0.0023 (4)	0.0093 (4)	−0.0008 (4)
O2	0.0370 (5)	0.0287 (4)	0.0239 (4)	0.0042 (4)	0.0191 (4)	0.0038 (3)
O3	0.0386 (7)	0.0270 (6)	0.0266 (6)	0.000	0.0223 (5)	0.000

### Geometric parameters (Å, °)

**Table:**

O1—C1	1.3660 (15)	C11—H11	0.9500
O1—H1A	0.86 (2)	C12—C13	1.3957 (15)
C1—C2	1.3884 (16)	C12—H12	0.9500
C1—C7	1.4040 (15)	C13—N2	1.3467 (13)
C2—C3	1.3821 (18)	C13—C14	1.4725 (15)
C2—H2	0.9500	C14—N3	1.2731 (14)
C3—C4	1.4012 (16)	C14—H14	0.9500
C3—H3	0.9500	N3—C15	1.4153 (13)
C4—C6	1.3885 (15)	C15—C16	1.4013 (14)
C4—C5	1.5083 (17)	C15—C21	1.4048 (14)
C5—H5A	0.9800	C16—C17	1.3870 (15)
C5—H5B	0.9800	C16—H16	0.9500
C5—H5C	0.9800	C17—C19	1.3998 (15)
C6—C7	1.4030 (16)	C17—C18	1.5072 (15)
C6—H6	0.9500	C18—H18A	0.9800
C7—N1	1.4139 (14)	C18—H18B	0.9800
N1—C8	1.2778 (14)	C18—H18C	0.9800
C8—C9	1.4721 (15)	C19—C20	1.3853 (15)
C8—H8	0.9500	C19—H19	0.9500
C9—N2	1.3445 (14)	C20—C21	1.3934 (15)
C9—C10	1.3985 (15)	C20—H20	0.9500
C10—C11	1.3827 (16)	C21—O2	1.3610 (13)
C10—H10	0.9500	O2—H2A	0.836 (17)
C11—C12	1.3857 (16)	O3—H3D	0.812 (16)
			
C1—O1—H1A	104.5 (13)	C12—C11—H11	120.6
O1—C1—C2	119.56 (10)	C11—C12—C13	119.14 (10)
O1—C1—C7	120.15 (10)	C11—C12—H12	120.4
C2—C1—C7	120.28 (11)	C13—C12—H12	120.4
C3—C2—C1	119.70 (10)	N2—C13—C12	122.72 (10)
C3—C2—H2	120.2	N2—C13—C14	118.71 (9)
C1—C2—H2	120.2	C12—C13—C14	118.57 (9)
C2—C3—C4	121.56 (11)	C9—N2—C13	117.41 (9)
C2—C3—H3	119.2	N3—C14—C13	122.07 (9)
C4—C3—H3	119.2	N3—C14—H14	119.0
C6—C4—C3	118.25 (11)	C13—C14—H14	119.0
C6—C4—C5	121.09 (10)	C14—N3—C15	119.91 (9)
C3—C4—C5	120.61 (10)	C16—C15—C21	118.89 (9)
C4—C5—H5A	109.5	C16—C15—N3	124.68 (9)
C4—C5—H5B	109.5	C21—C15—N3	116.42 (9)
H5A—C5—H5B	109.5	C17—C16—C15	121.93 (10)
C4—C5—H5C	109.5	C17—C16—H16	119.0
H5A—C5—H5C	109.5	C15—C16—H16	119.0
H5B—C5—H5C	109.5	C16—C17—C19	117.90 (10)
C4—C6—C7	121.31 (10)	C16—C17—C18	120.18 (10)
C4—C6—H6	119.3	C19—C17—C18	121.92 (10)
C7—C6—H6	119.3	C17—C18—H18A	109.5
C6—C7—C1	118.89 (10)	C17—C18—H18B	109.5
C6—C7—N1	126.84 (10)	H18A—C18—H18B	109.5
C1—C7—N1	114.20 (10)	C17—C18—H18C	109.5
C8—N1—C7	121.58 (10)	H18A—C18—H18C	109.5
N1—C8—C9	121.61 (10)	H18B—C18—H18C	109.5
N1—C8—H8	119.2	C20—C19—C17	121.49 (10)
C9—C8—H8	119.2	C20—C19—H19	119.3
N2—C9—C10	123.29 (10)	C17—C19—H19	119.3
N2—C9—C8	114.60 (9)	C19—C20—C21	120.05 (10)
C10—C9—C8	122.07 (10)	C19—C20—H20	120.0
C11—C10—C9	118.58 (10)	C21—C20—H20	120.0
C11—C10—H10	120.7	O2—C21—C20	118.06 (9)
C9—C10—H10	120.7	O2—C21—C15	122.20 (10)
C10—C11—C12	118.84 (10)	C20—C21—C15	119.72 (9)
C10—C11—H11	120.6	C21—O2—H2A	112.5 (10)
			
O1—C1—C2—C3	178.93 (11)	C11—C12—C13—C14	179.63 (10)
C7—C1—C2—C3	−0.42 (18)	C10—C9—N2—C13	−1.22 (16)
C1—C2—C3—C4	−0.16 (18)	C8—C9—N2—C13	176.55 (9)
C2—C3—C4—C6	0.66 (18)	C12—C13—N2—C9	1.05 (15)
C2—C3—C4—C5	−176.69 (12)	C14—C13—N2—C9	−178.70 (9)
C3—C4—C6—C7	−0.61 (17)	N2—C13—C14—N3	−15.80 (16)
C5—C4—C6—C7	176.73 (11)	C12—C13—C14—N3	164.44 (11)
C4—C6—C7—C1	0.06 (17)	C13—C14—N3—C15	−177.17 (9)
C4—C6—C7—N1	−176.84 (11)	C14—N3—C15—C16	16.85 (16)
O1—C1—C7—C6	−178.88 (10)	C14—N3—C15—C21	−164.27 (10)
C2—C1—C7—C6	0.47 (17)	C21—C15—C16—C17	1.35 (16)
O1—C1—C7—N1	−1.61 (16)	N3—C15—C16—C17	−179.79 (10)
C2—C1—C7—N1	177.74 (10)	C15—C16—C17—C19	−0.49 (16)
C6—C7—N1—C8	−3.83 (18)	C15—C16—C17—C18	−179.90 (10)
C1—C7—N1—C8	179.15 (10)	C16—C17—C19—C20	−0.50 (16)
C7—N1—C8—C9	176.17 (10)	C18—C17—C19—C20	178.90 (10)
N1—C8—C9—N2	176.83 (10)	C17—C19—C20—C21	0.60 (17)
N1—C8—C9—C10	−5.37 (17)	C19—C20—C21—O2	178.48 (10)
N2—C9—C10—C11	0.45 (17)	C19—C20—C21—C15	0.29 (16)
C8—C9—C10—C11	−177.16 (10)	C16—C15—C21—O2	−179.34 (10)
C9—C10—C11—C12	0.52 (17)	N3—C15—C21—O2	1.71 (15)
C10—C11—C12—C13	−0.68 (17)	C16—C15—C21—C20	−1.24 (15)
C11—C12—C13—N2	−0.12 (17)	N3—C15—C21—C20	179.82 (10)

### Hydrogen-bond geometry (Å, °)

**Table:**

*D*—H···*A*	*D*—H	H···*A*	*D*···*A*	*D*—H···*A*
O2—H2A···O3	0.836 (17)	1.960 (17)	2.7592 (12)	159.6 (15)
O2—H2A···N3	0.836 (17)	2.347 (15)	2.7624 (12)	111.3 (12)
O1—H1A···N1	0.86 (2)	2.120 (19)	2.6722 (13)	121.7 (16)
O3—H3D···N2	0.812 (16)	2.187 (16)	2.9865 (12)	168.0 (16)
O3—H3D···N3	0.812 (16)	2.569 (16)	3.0142 (9)	115.9 (14)
